# miR-3960 from Mesenchymal Stem Cell-Derived Extracellular Vesicles Inactivates SDC1/Wnt/*β*-Catenin Axis to Relieve Chondrocyte Injury in Osteoarthritis by Targeting PHLDA2

**DOI:** 10.1155/2022/9455152

**Published:** 2022-08-25

**Authors:** Peng Ye, Zhanhu Mi, Daihao Wei, Pengcheng Gao, Mei Ma, Haibo Yang

**Affiliations:** Department of Orthopedic Trauma, General Hospital of Ningxia Medical University, Yinchuan 750004, China

## Abstract

Osteoarthritis (OA) is a serious disease of the articular cartilage characterized by excessive inflammation. Lately, mesenchymal stem cell- (MSC-) derived extracellular vesicles (EVs) have been proposed as a novel strategy for the treatment of OA. We aimed to investigate the effects of EV-encapsulated miR-3960 derived from MSCs on chondrocyte injury in OA. The cartilage tissues from OA patients were collected to experimentally determine expression patterns of miR-3960, PHLDA2, SDC1, and *β*-catenin. Next, luciferase assay was implemented to testify the binding affinity among miR-3960 and PHLDA2. EVs were isolated from MSCs and cocultured with IL-1*β*-induced OA chondrocytes. Afterwards, cellular biological behaviors and levels of extracellular matrix- (ECM-) related protein anabolic markers (collagen II and aggrecan), catabolic markers (MMP13 and ADAMTS5), and inflammatory factors (IL-6 and TNF-*α*) in chondrocytes were assayed upon miR-3960 and/or PHLDA2 gain- or loss-of-function. Finally, the effects of miR-3960 contained in MSC-derived EVs in OA mouse models were also explored. MSCs-EVs could reduce IL-1*β*-induced inflammatory response and extracellular matrix (ECM) degradation in chondrocytes. miR-3960 expression was downregulated in cartilage tissues of OA patients but enriched in MSC-derived EVs. miR-3960 could target and inhibit PHLDA2, which was positively correlated with SDC1 and Wnt/*β*-catenin pathway activation. miR-3960 shuttled by MSC-derived EVs protected against apoptosis and ECM degradation in chondrocytes. In vivo experiment also confirmed that miR-3960 alleviated chondrocyte injury in OA. Collectively, MSC-derived EV-loaded miR-3960 downregulated PHLDA2 to inhibit chondrocyte injury via SDC1/Wnt/*β*-catenin.

## 1. Introduction

Osteoarthritis (OA) is the most universal joint disease in the world, with the increasing incidence and prevalence related to age [1]. Therefore, this disorder gives rise to a huge and augmenting health burden due to its significant impact on the affected individuals, healthcare system, and broader socioeconomic costs [2]. As the only cell type in articular cartilage, apoptosis of chondrocytes and cytokine production exert pivotal function on the pathological process of OA; reducing chondrocyte apoptosis and inflammation may be effective to alleviate OA symptoms [3]. The potential of mesenchymal stem cells (MSCs) to enhance the chondrogenic differentiation renders them as new therapeutic strategies for OA [4]. A recent study has demonstrated that MSC-derived extracellular vesicles (EVs) act as a novel cell-free therapy approach for treating OA [5]. The complex molecular mechanisms underlying the action of MSC-derived EVs in OA-related chondrocyte injury remain to be further elucidated.

The membrane-derived vesicles, EVs, are secreted by various cells under normal and pathological conditions [6] and have emerged as an important cell-cell communication entity in physiological and pathological processes by acting as a carrier of many biomolecules, including microRNAs (miRNAs), which are involved in many biological processes that regulate tissue homeostasis and physiopathology of multiple diseases, including OA [7]. Existing literature has confirmed that EVs delivering miRNAs play a vital role in OA treatment [8]. miRNAs, highly conserved small noncoding RNAs with 21-23 nucleotides in length, could regulate the expression of relevant genes by binding to 3′untranslated region (3'UTR) [9, 10]. Mounting evidences indicate that aberrant expression of miRNAs is involved in inflammatory diseases, including OA [11, 12]. For instance, miR-152 could protect articular cartilage to suppress OA [13]. Strikingly, miR-3960 is reported to participate in the osteogenic transdifferentiation of vascular smooth muscle cells [14], yet few studies have reported its roles in chondrocyte injury in OA. Moreover, the bioinformatic analysis in the current study predicted that miR-3960 could target PHLDA2. PHLDA2, as an important gene in the imprinted gene domain of 11p15.5, is an important factor to destroy heterochromatin and elevate the expression of PHLDA2 [15]. It has been reported that PHLDA2, as a new biomarker for hypertrophic chondrocytes, is implicated in the pathology of OA [16]. Based on the before-mentioned findings, we proposed a hypothesis that the transfer of miR-3960 via MSC-derived EVs might alter chondrocyte injury in OA. Hence, in vitro model of interleukin-1*β*-(IL-1*β*-) treated chondrocytes was developed for exploration at cellular levels. In addition to that, we also established a mouse model of OA for validation.

## 2. Materials and Methods

### 2.1. Ethical Approval

All study protocols were approved by the Ethics Committee of General Hospital of Ningxia Medical University with written informed consent gained from all participating patients. All animal experiments were implemented in strict compliance with the recommendations of the Guide for the Care and Use of Laboratory Animals issued by the National Institutes of Health and granted by the Institutional Animal Care and Use Committee of Ningxia Medical University.

### 2.2. Patient Enrollment

The cartilage tissues of the knee joint (normal control) were excised from 20 patients with traumatic amputation in the absence of rheumatoid arthritis or OA (7 males and 13 females; aged 50-72 years; with a mean age of 62.55 years). Meanwhile, cartilage tissues were isolated from 20 OA patients (8 males and 12 females; aged 48-71 years; with a mean age of 62.30 years) following a total knee arthroplasty. All OA patients were diagnosed by imaging, laboratory examination, signs, and symptoms, excluding any factors that may affect disease progression.

### 2.3. Isolation, Culture, and Identification of Chondrocytes

Chondrocytes were isolated from the knee joint of four C57BL/6 mice. Cartilage of the knee joint was isolated from 24 C57BL/6 J male mice (aging 8 weeks and weighing 20 g ± 25 g) under aseptic condition and placed in the culture dish. After washing with PBS, the cartilage tissues were cut into small pieces (<1 mm × 1 mm × 1 mm) using ophthalmic scissors. The tissues were digested with 0.2% Type II collagenase (Invitrogen, Carlsbad, CA) along with 0.25% trypsin (Sigma, St Louis, MO) in a dish at 37°C for 30 min. After that, cell suspension was filtered by a mesh net 150 and centrifuged at 1000 r/min (3-5 min). The pellets were cultured in a Dulbecco's Modified Eagle's Medium (DMEM; Gibco, Grand Island, NY) replenished with 10% fetal bovine serum (FBS; Gibco), 1% penicillin, and 1% streptomycin (Gibco). Medium plate containing chondrocytes was cultured in a 5% CO_2_ incubator with saturated humidity for 24 h with the medium renewed every 2 days. Cells were observed under the inverted microscope. Following 80–90% confluence, the chondrocytes were passaged with 2.5 g/L trypsin and 0.02% ethylenediaminetetraacetic acid (EDTA).

After primary culture and subculture, chondrocytes stained with hematoxylin and eosin (H&E) were visualized under the inverted microscope every 2 days and photographed.

Chondrocytes were seeded on the aseptic cover glass in a 6-well plate and fixed with 40 g/L paraformaldehyde (20 min) and 75% ethanol (15 min), followed by staining with 1% toluidine blue (G2543, Solarbio, Beijing, China) at 60°C for 2-3 h. After conventional treatments, the images of stained chondrocytes were captured under the microscope.

After seeding and incubation, the primary and passaged mouse chondrocytes were fixed with 40 g/L paraformaldehyde for 20 min and sealed for 2 h. Another incubation of 3-5 h was conducted with the primary antibody, followed by incubation with the fluorescence secondary antibody in a dark room, staining with 4',6-diamidino-2-phenylindole (DAPI), and microscopic observation. PBS was utilized as the negative control (NC) instead of the primary antibody (Figure S1).

### 2.4. Construction of OA Model In Vitro

The chondrocytes at the 3^rd^ to 6^th^ passage were adopted. Upon 80% cell confluency, the chondrocytes were administered with interleukin-1*β* (IL-1*β*, 10 ng/mL; Peprotech, NJ) for 24 h to mimic an OA-like state in vitro.

### 2.5. Extraction and Characterization of MSCs-EVs

The mouse bone marrow-derived MSCs (BMSCs) were available from the American type culture collection (Manassas, VA). MSCs were cultured in DMEM deprived of serum for 2 days. Then, the cell supernatant was centrifuged at 8000 × *g* for 30 min to remove dead cells and debris; then, the small cell debris was filtered using a 0.22 *μ*m filter. The supernatant was inhaled into the ultrafiltration tube and centrifuged at 100, 000 × *g* for 1 h at 4°C. The harvested MSCs were purified and then resuspended in PBS, followed by the second ultracentrifugation under the same conditions. The precipitate was stored at -80°C for the following experiments.

Next, 10 *μ*L MSCs-EVs were added to the sealing film, and the copper mesh Formvar membrane was placed face down on the suspension. The 2-3 copper nets were prepared for each MSCs-EV sample, which was covered to allow absorption by the copper mesh in a dry environment. A total of 100 *μ*L PBS were added to the sealing film. The mesh (Formvar film face down) was placed on PBS drops with tweezers for 5 min and washed twice, on 50 *μ*L 1% glutaraldehyde drops for 5 min, on 50 *μ*L uranyl oxalate droplet for 5 min, and 50 *μ*L methylcellulose UA drops for 10 min on ice. The mesh was next removed, and the excess liquid was gently discarded using the filter paper, leaving a thin layer of methyl cellulose membrane, which was dried in air for 10 min. The images of dried copper mesh were captured at 100KeV for electron microscopic observation (HT7830, Hitachi, Tokyo, Japan). The surface marker proteins of EVs were identified by immunoblotting. EV particles were dissolved in radio immunoprecipitation assay (RIPA) buffer and quantified by bicinchoninic acid (BCA) protein analysis kit (a53226, Thermo Fisher Scientific, Rockford, IL). Antibodies used in immunoblotting of EVs were as follows: CD9 (ab92726, 1: 1000, Abcam, Cambridge, UK); CD63 (ab134045, 1: 1000, Abcam); Calnexin (ab22595, 1: 100, Abcam); and TSG 101 (ab125011, 1: 1000, Abcam).

Nanoparticle tracking analysis (NTA) was performed to analyze the particle size of EVs. EVs were resuspended and mixed in 1 mL PBS, with filtered PBS as the control. Then, diluted EVs were loaded onto the NanoSight LM10 instrument (NanoSight Ltd., Minton Park, UK) to measure the particle size under the condition of 23.75 ± 0.5°C for 60 s.

All the abovementioned procedures complied with the International Society for Extracellular Vesicles and update of the MISEV2018 guidelines [17].

### 2.6. EV Uptake Assay

The isolated MSC-EVs were labeled with red fluorescent dye PKH26 (MINI26-1KT, Sigma) at an ambient temperature for 5 min. After centrifugation at 100,000 × *g* for 90 min, the EVs were suspended in the culture medium, which were cultured with chondrocytes at 37°C for 12 h, fixed in 4% paraformaldehyde, and then dyed utilizing 0.1 g/mL DAPI (C1002, Beyotime, Shanghai, China) for 5 min. SH-SY5Y cells were visualized under a fluorescence microscope (BX53; Olympus, Tokyo, Japan). Hoechst 33342 staining (10 *μ*g/mL, C1025, Beyotime) was implemented for nuclear staining. Cytoskeleton was dyed with Actin-Tracker Green (Beyotime). The uptake of EV was recorded utilizing a confocal laser scanning microscope (Zeiss LSM710, Jena, Germany).

### 2.7. Cell Transfection

Chondrocytes or MSCs were trypsinized, seeded into a 6-well plate (1 × 10^5^ cells/well), and subsequently incubated for 24 h. Upon cell confluency reached about 75%, transfection was implemented according with the instructions of Lipofectamine 2000 (Invitrogen). Cells were manipulated with mimic NC/miR-3960 mimic, inhibitor-NC/miR-3960 inhibitor, control shRNA (sh), sh-PHLDA2, sh-SDC1, oe-NC, oe-SDC1, mimic NC+vector, mimic NC+PHLDA2 vector, miR-3960 mimic+PHLDA2 vector, and agomir NC/miR-3960 agomir. After incubation for 36 h, cells were cultured with IL-1*β* and 20 mM LiCl (Wnt/*β*-catenin pathway agonist) for 24 h [18]. The expression plasmid (50 ng/mL) was purchased from GenePharma (Shanghai, China).

### 2.8. Cell Proliferation Assay

Cells were seeded into 96-well plates (1 × 10^3^ cells/well) and then cultured in 100 *μ*L medium containing 10% FBS. Then, the cells were cultured for 1-5 days, and the number of cells was determined by Cell Counting Kit-8 (CCK8; K1018, Apexbio, Boston, MA). Then, 10 *μ*L CCK8 solution was added to each well of the plate (5 parallel wells/group) and incubated (37°C;4 h). Optical density (OD) value at 450 nm was obtained, and OD values on the 1^st^, 3^rd^, 5^th^, and 7^th^ day were recorded.

### 2.9. Cell Apoptosis Assay

Annexin V-allophycocyanin apoptosis detection kit (556547, BD pharmacingen, San Jose) was adopted to detect chondrocyte apoptosis. Falcon tubes were taken, and NC tubes and tube numbers of specimens were arranged according to the sequence of specimens. Chondrocyte suspension (1 × 10^6^ cells/mL) was prepared with 1×Binding buffer and 100 *μ*L of which was added into Falcon tube. Next, annexin V and nucleic acid dye were also added, mixed gently, and reacted in the dark at ambient temperature (20-25°C; 15 min). Annexin V-biotin reagent was used. Cells were washed with 1×binding buffer once to remove the supernatant. Then, Sav-FITC (0.5 *μ*g) was dissolved in 1×binding buffer (100 *μ*L), which was added into the cell tube and mixed gently. Next, cells were dyed with 5 *μ*L propidium iodide in the dark at ambient temperature for 15 min. Each test tube was added with 400 *μ*L of 1×binding buffer, and apoptotic cells were calculated by means of FACScan flow cell flow system (Becton Dickinson, San Diego, CA, USA) within 1 h.

### 2.10. Dual-Luciferase Reporter Gene Assay

Dual-luciferase reporter gene vectors of PHLDA2 3'UTR and mutant plasmid with miR-3960 binding site (PmirGLO-PHLDA2-WT and PmirGLO-PHLDA2-MUT) were designed. Luciferase reporter plasmids WT and MUT with miR-3960 mimic or NC-mimic were cointroduced into 293T cells. After transduction for 48 h, the cells were centrifuged at 12000 × *g* for 1 min to collect the supernatant. The luciferase activity was detected using Dual-Luciferase® Reporter Assay System (E1910, Promega, Madison, WI, USA). Each cell sample was added with 100 *μ*L firefly or Renilla luciferase working solution to detect firefly or Renilla luciferase. Relative luciferase activity = firefly luciferase activity/Renilla luciferase activity.

### 2.11. Cy3 Fluorescent Labeling

MSCs were trypsinized and then resuspended with DMEM containing 10% FBS. The cell concentration was adjusted to 1 × 10^6^ cells/well. Cy3-labeled miR-3960 (miR-3960-Cy3) (GenePharma) was transduced into MSCs utilizing Lipofectamine 2000 (11668019, Invitrogen) to identify the transfer of miR-3960 into MSCs via EVs. MSCs expressing Cy3-miR-3960 were seeded into 6-well plates and cocultured with chondrocytes in Transwell chamber for 2-4 days. Subsequently, Hoechst 33342 staining was implemented. Finally, the chondrocyte nucleus and Cy3-labeled miR-3960 were observed under the confocal microscope.

### 2.12. Reverse Transcription-Quantitative Polymerase Chain Reaction (RT-qPCR)

TRIzol (Product No. 16096020, Thermo Fisher Scientific) was adopted to extract total RNA. For mRNA detection, reverse transcription kit (RR047A, Takara Bio Inc., Otsu, Shiga, Japan) was utilized to produce cDNA. For miRNA, PolyA tailing detection kit (B532451, Shanghai Sangon Biotechnology Co. Ltd., Shanghai, China) was utilized (containing universal polymerase chain reaction (PCR) primer R and universal PCR primer R of U6) to obtain the cDNA of the miRNA with PolyA tail. SYBR® Premix Ex Taq™ II kit (DRR081, Takara Biotechnology Ltd., Dalian, China) was adopted for sample loading, and RT-qPCR was performed in a real-time fluorescent quantitative PCR instrument (ABI 7500, ABI, Foster City, CA, USA). U6 and GAPDH served as an internal control for miRNA and mRNA, respectively, while syn-cel-miR-39 as an endogenous control for data normalization of miRNA in EVs. The primer sequences of genes are listed in Table S1. The 2^-*ΔΔ*Ct^ method was utilized to quantify relative expression levels of target genes.

### 2.13. Immunoblotting

The total protein was extracted from tissues and cells using RIPA (P0013B, Beyotime Biotechnology). Nuclear and cytoplasmic proteins were extracted by nucleoprotein and cytoplasmic protein extraction kit (P0028, Beyotime Biotechnology). Protein concentration was detected by BCA kit (A53226, Thermo Fisher Scientific). The protein was separated by sodium dodecyl sulfate-polyacrylamide gel electrophoresis and electrotransferred onto a polyvinylidene fluoride membrane (IPVH85R, Millipore, Darmstadt, Germany). Membrane was blocked with 5% bovine serum albumin (BSA) at ambient temperature for 1 h and then incubated overnight at 4°C with the following primary antibodies: rabbit polyclonal anti- collagen II (ab34712, 1: 5000, Abcam); aggrecan (13880-1-AP, 1: 400, Proteintech, Wuhan, China); MMP13 (ab39012, 1: 4000, Abcam); ADAMTS5 (ab41037, 1 : 10, Abcam); *β*-catenin (ab224803, 1: 400, Abcam); lamin B (ab223013, 1: 1000, Abcam); and GAPDH (ab181602, 1: 10000, Abcam). Subsequently, the membrane was washed and incubated with the HRP-labeled goat antirabbit secondary antibody immunoglobulin G (IgG) (ab6721, 1: 5000, Abcam) at ambient temperature for 1 h. Membrane was developed by chemiluminescence instrument. The results were analyzed by the 1.48 u ImageJ software (V1.48, National Institutes of Health, Maryland, USA). The relative protein expression was expressed as the ratio of the gray value of protein to be tested to that of internal reference (GAPDH).

### 2.14. ELISA

The supernatant of chondrocytes was collected. In brief, after dilution to 1-10 *μ*g/mL protein content with 0.05 M pH 9 carbonate-coated buffer, 0.1 mL of antibody was added into the reaction well of each polystyrene plate at 4°C overnight. The next day, the solution was discarded. A certain amount of diluted sample (0.1 mL) was added into the coated reaction well for 1 h of incubation (37°C). Each well was added with freshly diluted enzyme-labeled antibody (0.1 mL) and TMB substrate solution (0.1 mL; 37°C) for 10-30 min of reaction. Finally, 0.05 mL of 2 M sulfuric acid was utilized to terminate the reaction followed by OD value measurement at 450 nm by ELISA.

### 2.15. Animal Experiments

A total of 32 C57BL/6 J male mice (aging 8 weeks and weighing 20 g ± 5 g) were used for in vivo experiments. Among them, 24 mice were operated, and the remaining 8 mice sham-operated to serve as control. According to Farnaghi et al. [19], OA mouse models were established by standard operation procedure. The animals were subjected to intraperitoneal injection of 3% pentobarbital for anesthesia. The mice with supine position were fixed on the operating table. Skin inside the bilateral knee joints was clamped with ophthalmic forceps, and then the incision no longer than 1 cm was cut longitudinally. The medial collateral ligament was exposed in the center of the operative field of vision, resulting in dislocation of the patella. The medial collateral ligament at the joint junction was cut transversely with ophthalmic scissors to form a 5 *μ*m incision. With ophthalmic forceps, the joint capsule at the upper and lower edge of meniscus was gently cut at incision of medial collateral ligament until the translucent meniscus was exposed. The medial meniscus was hooked out with self-made periodontal probe and cut with ophthalmic scissors. The meniscus and medial collateral ligament were left at the incision. For the sham-operated mice, after anesthesia, the skin inside the bilateral knee joints was clamped with ophthalmic forceps, and then the incision no longer than 1 cm was cut longitudinally, but the medial collateral ligament and medial meniscus were not cut off. After the patella was reset and the wound was cleaned with normal saline, incision was sutured layer by layer. To avoid the possible influence of individual differences, the mice were randomly assigned into 4 groups with 8 mice/group: 8 mice were sham-operated (after anesthesia, only the medial collateral ligament was exposed in the center of the surgical field but not cut off); 8 mice were induced with OA-like changes (the transversely sheared medial collateral ligament at the joint junction and the incised capsule at the upper and lower edges of the meniscus resulted in OA changes of the knee joint); 8 OA mice were injected with MSCs-EVs-agomir-NC (mice rendered with OA-like changes were injected with 100 *μ*g/mL of MSCs-EVs-agomir-NC); and 8 OA mice were injected with MSCs-EVs-miR-3960 agomir (mice rendered with OA-like changes were injected with 100 *μ*g/mL MSCs-EVs-miR-3960 agomir). In detail, at the 4^th^ week of OA induction, 10 *μ*L MSCs-EVs or sterile normal saline were injected into the articular capsule for 3 weeks (once a week). After 7 weeks of OA induction, the mice were sacrificed and the osteoarticular tissues were taken for follow-up experiments.

### 2.16. Hematoxylin and Eosin (H&E) Staining

Sections of the knee-joint cartilage tissues were selected, dried, and fixed at ambient temperature for 30 s. The sections were stained with hematoxylin for 60 s, differentiated with alcohol hydrochloride for 3 s, stained with eosin for 3 min, and dehydrated with ethanol (70%, 80%, and 95%) and anhydrous ethanol for 5 min. The sections were sealed with gum. At last, the morphological observation of cartilage tissues was implemented under the microscope (BX63).

### 2.17. Alcian Blue Staining

Paraffin-embedded mouse knee-joint cartilage tissues were cut into serial sections. The paraffin-embedded sections were dehydrated with gradient ethanol (70%, 80%, 95% ethanol, and anhydrous ethanol), washed with xylene, and rinsed with deionized water. Next, the sections were stained with alcian blue for 30 min and immersed in 0.5% glacial acetic acid for 5 s. Finally, the sections were subjected to conventional procedures (dehydration, clearing, and mounting), followed by morphological observation of cartilage tissues under the light microscope.

### 2.18. Terminal Deoxynucleotidyl Transferase-Mediated Nick End Labeling (TUNEL) Assay

Chondrocyte apoptosis was quantified utilizing a TUNEL Kit (11684817910, Roche, Mannheim, Germany). The mouse knee-joint cartilage tissues were sectioned, and the paraffin-embedded sections were routinely dewaxed to water and immersed in 3% H_2_O_2_ methanol solution for 30 min at ambient temperature to block peroxidase. After incubation with proteinase K solution at 37°C for 30 min, the cartilage tissue sections were allowed to react in 0.1% Triton X-100 and 0.1% sodium citrate solution at ambient temperature for 20 min. The sections were incubated with newly prepared TUNEL reaction mixture (prepared according to the instructions of TUNEL Kit) at 37°C for 2 h, followed by reaction with 3% BSA at ambient temperature in wet box for 20 min. The sections were added with the transformed peroxidase solution at 37°C for 30 min, followed by developing with diaminobenzidine. The number of TUNEL-positive cells (brownish yellow granules in the nuclei) in each field was microscopically counted with the application of BI-2000 image analysis system, and that in five fields of each section was averaged.

### 2.19. Safranin O-Fast Green Staining

Paraffin-embedded cartilage sections were usually dewaxed to water, followed by staining with hematoxylin for 10-30 min, and differentiated with 1% hydrochloric acid ethanol solution for 210 s until the slices being light red. Next, the sections were rinsed and dehydrated (50%, 70%, and 80% ethanol). After counterstaining, sections were dehydrated in 95% ethanol, and the excessive dye was removed by anhydrous ethanol for 3-5 min and sealed with neutral gum. Through the analysis of microscopic images, cartilage degradation was quantified by two independent observers in a double-blind fashion using the International Association for The Study of Osteoarthritis (OARSI) grading system [20].

### 2.20. Pain Assessment

The mechanical and thermal allodynia assessments were implemented by measuring the paw withdrawal threshold (PWT) and thermal paw withdrawal latency (PWL) prior to modeling, 1, 2, 4, and 6 weeks after modeling. For PWT measurement, center of the mouse hind paw was vertically stimulated by von Frey fibers (Aesthesio, Italy) with increasing intensity. Quickly flinching or licking was indicative of a positive withdrawal reaction, and a stimulation at an adjacent reduced intensity was given accordingly. If negative withdrawal reaction occurs, a stimulation at an adjacent increased intensity will be given till the positive withdrawal reaction. PWT was expressed by the von Frey fibers value when 3 positive withdrawal reactions occurred among 5 consecutive stimuli. An automatic Plantar test (Hargreaves Apparatus, Italy) was adopted to stimulate the hind paw of the mouse for PWL measurement. The mouse was placed on the clear glass and covered with a clear cap, and the hind paw surface was exposed to infrared heat source. The time interval between onset of thermal stimulation and paw withdrawal was defined as PWL. Each mouse was recorded 3 times with an interval over 5 min between two measurements.

### 2.21. Statistical Analysis

Statistical analysis was performed with SPSS 21.0 statistical software (SPSS, Inc., IBM, Armonk, NY). All measurement data are presented as mean ± standard deviation. Data between two groups conforming to normal distribution were compared utilizing unpaired *t*-test, whereas the skewed data were analyzed by rank sum test. Enumeration data comparison was conducted by *x*^2^ test. For multiple independent groups, one-way analysis of variance (ANOVA) with Tukey's post hoc test was utilized. The comparison of data at different time points was analyzed using repeated measurement ANOVA with Tukey's post hoc analysis. The correlations among miR-3960, PHLDA2, and SDC1 were analyzed by Pearson's correlation coefficient. Values of *p* < 0.05 were considered significant.

## 3. Results

### 3.1. MSC-Derived EVs Inhibit Chondrocyte Injury in OA

**Figure 1:**
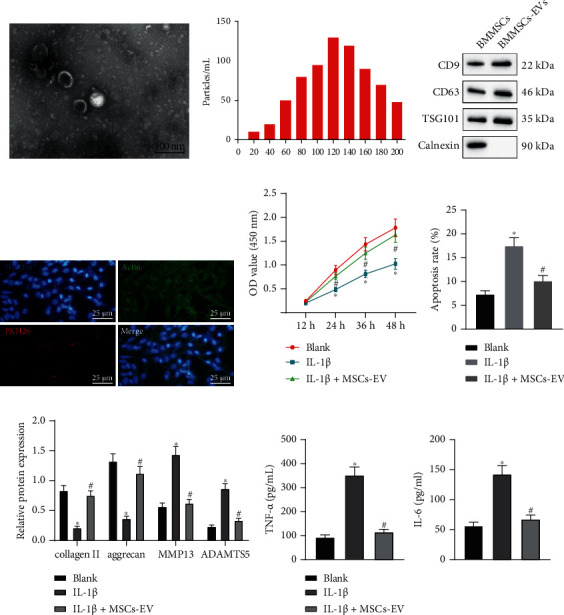
MSCs-EVs suppresses IL-1*β*-induced chondrocyte inflammation and ECM degradation in OA. (a) The morphology of EVs observed under the TEM (scale bar = 100 nm). (b) The distribution of EVs analyzed by NTA. (c) Protein levels of CD9, CD63, TSG101, and Calnexin measured by immunoblotting. (d) The uptake of EVs by chondrocytes observed under the microscope (scale bar = 25 *μ*m). Chondrocytes were treated with IL-1*β* and/or MSCs-EVs. (e) Cell proliferation assessed by CCK8 assay. (f) Apoptosis assessed by flow cytometry. (g) Protein levels of collagen II, aggrecan, MMP13, and ADAMTS5 in chondrocytes measured by immunoblotting. (h) Levels of IL-6 and TNF-*α* in chondrocytes measured by ELISA. ∗*p* < 0.05 vs. BMMSCs or chondrocytes without treatment, ^#^*p* < 0.05 vs. chondrocytes treated with IL-1*β*. Data are shown as the mean ± standard deviation of three technical replicates. Data between two groups were compared by independent *t*-test. Data comparisons among multiple groups were analyzed by the one-way ANOVA with Tukey's post hoc test, and comparison of data at different time points was analyzed using repeated measurement ANOVA with Tukey's post hoc test.

First of all, the EVs were isolated from MSCs using ultracentrifugation. The isolated MSCs-EVs were round or oval membranous vesicles shown by transmission electron microscopic observation (Figure 1(a)), and NTA exhibited their diameter within 30-150 nm (Figure 1(b)). Immunoblotting additionally showed the abundance of CD9, CD63, and TSG101 but the absence of calnexin in EVs (Figure 1(c)), which confirmed that EVs were successfully extracted. Chondrocytes were incubated with PKH26- (red-) labeled EVs for 24 h. The microscopic images displayed the significant internalization of PKH26-labeled EVs by chondrocytes (Figure 1(d)).

To explore the effect of MSCs-EVs on chondrocytes, the chondrocyte was induced by 10 ng/mL IL-1*β* to induce in vitro OA-like changes, and then, chondrocytes were treated with 100 *μ*g/mL MSCs-EVs. CCK8 assay and flow cytometric data displayed that IL-1*β* treatment caused inhibited cell proliferation and promoted apoptosis, which were witnessed to be reversed upon treatment with MSCs-EVs (Figures 1(e) and 1(f)). Immunoblotting and ELISA presented decreased levels of anabolic markers (collagen II and aggrecan) and increased levels of catabolic markers (MMP13 and ADAMTS5), IL-6, and TNF-*α* in chondrocytes treated with IL-1*β*. However, MSCs-EVs contributed to reversing IL-1*β*-induced affects on the aforementioned molecules (Figures 1(g) and 1(h)). These findings suggested an anti-inflammatory effect of MSCs-EVs on chondrocytes and its potential to prevent ECM degradation.

### 3.2. MSCs-EV-Encapsulated miR-3960 Inhibits Chondrocyte Injury in OA

**Figure 2:**
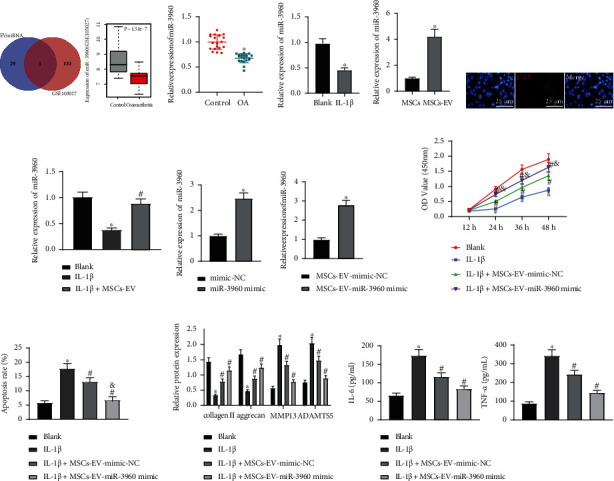
MSCs-EVs carrying miR-3960 inhibits chondrocyte injury in OA. (a) Venn diagram of 134 downregulated miRNAs (red circle) in GSE105027 dataset and the top 30 miRNAs enriched in MSCs-EVs (blue circle) and miR-3960 expression in GSE105027 dataset. (b) miR-3960 expression in the cartilage tissues of OA patients (*n* = 20) and patients with traumatic amputation (*n* = 20) determined by RT-qPCR. (c) miR-3960 expression in the IL-1*β*-treated chondrocytes determined by RT-qPCR. (d) The expression of miR-3960 in MSCs-EVs was determined by qRT-PCR (*p* < 0.05 vs. that in MSCs). (e) Transfer of Cy3-labeled miR-3960 into chondrocytes via MSCs-EVs (scale bar = 25 *μ*m). (f) miR-3960 expression in the chondrocytes treated with IL-1*β*+MSCs-EVs determined by RT-qPCR. (g) miR-3960 expression in the chondrocytes treated with miR-3960 mimic determined by RT-qPCR. (h) miR-3960 expression in the chondrocytes treated with IL-1*β*+MSCs-EVs-miR-3960 mimic determined by RT-qPCR. Chondrocytes were treated with IL-1*β* and/or MSCs-EVs-miR-3960 mimic. (i) Cell proliferation assessed by CCK8 assay. (j) Apoptosis assessed by flow cytometry. (k) Protein levels of collagen II, aggrecan, MMP13, and ADAMTS5 in chondrocytes measured by immunoblotting. (l) Levels of IL-6 and TNF-*α* in chondrocytes measured by ELISA. ^∗^*p* < 0.05 vs. patients with traumatic amputation, chondrocytes without treatment, chondrocytes treated with mimic-NC, or chondrocytes treated with MSCs-EVs-mimic-NC; ^#^*p* < 0.05 vs. chondrocytes treated with IL-1*β*; & *p* < 0.05 vs. chondrocytes treated with IL-1*β*+MSCs-EVs-mimic-NC. Data are shown as the mean ± standard deviation of three technical replicates. Data between two groups were compared by independent *t*-test. Data comparisons among multiple groups were analyzed by the one-way ANOVA with Tukey's post hoc test, and comparison of data at different time points was analyzed using repeated measurement ANOVA with Tukey's post hoc test.

The study's focus was next shifted onto the significance of miRNAs in EVs. We screened the first 30 miRNAs (blue circle) secreted by EVs from human MSCs through EVmiRNA database. The 134 downregulated miRNAs (red circle) in GSE105027 and the top 30 miRNAs in MSCs-EVs were analyzed by Venn, which showed that miR-3960 was the only intersection. Moreover, GSE105027 database (miRNA expression in serum of 12 patients with OA and 12 healthy individuals) [21] showed the downregulation of miR-3960 in OA samples (Figure 2(a)). RT-qPCR displayed decreased miR-3960 expression in the OA cartilage tissues and IL-1*β*-treated chondrocytes (Figures 2(b) and 2(c)). The qRT-PCR results revealed that miR-3960 was highly expressed in MSCs-EV (Figure 2(d)). Chondrocytes were incubated with Cy3-labeled miR-3960 shuttled by MSCs-EVs, and it was found that chondrocytes exhibited red fluorescence, and Cy3-labeled miR-3960 was internalized into chondrocytes (Figure 2(e)). Furthermore, MSCs-EV treatment elevated miR-3960 expression in the chondrocytes treated with IL-1*β* (Figure 2(f)). It was suggested that MSCs-EVs could carry miR-3960 into chondrocytes.

After overexpression of miR-3960 in MSCs, MSCs-EVs with overexpressed miR-3960 were extracted to explore the significance of MSCs-EV-encapsulated miR-3960 in chondrocytes (Figure 2(g)). RT-qPCR presented enhancement of miR-3960 expression in chondrocytes treated with MSCs-EVs-miR-3960 mimic (Figure 2(h)). Furthermore, MSCs-EVs-mimic-NC treatment promoted cell proliferation and repressed apoptosis in the IL-1*β*-treated chondrocytes, which effects were potentiated by the treatment of MSCs-EVs-miR-3960 mimic (Figures 2(i) and 2(j)). Additionally, MSCs-EVs-mimic-NC led to reduced collagen II and aggrecan levels and elevated MMP13, ADAMTS5, IL-6, and TNF-*α* expressions in the chondrocytes treated with IL-1*β*, while MSCs-EVs-miR-3960 mimic induced significant effects superior to MSCs-EVs-mimic-NC (Figures 2(k) and 2(l)). It can be concluded that miR-3960 shuttled by MSCs-EVs suppressed chondrocyte inflammation and ECM degradation.

### 3.3. miR-3960 Targets and Inhibits PHLDA2

**Figure 3:**
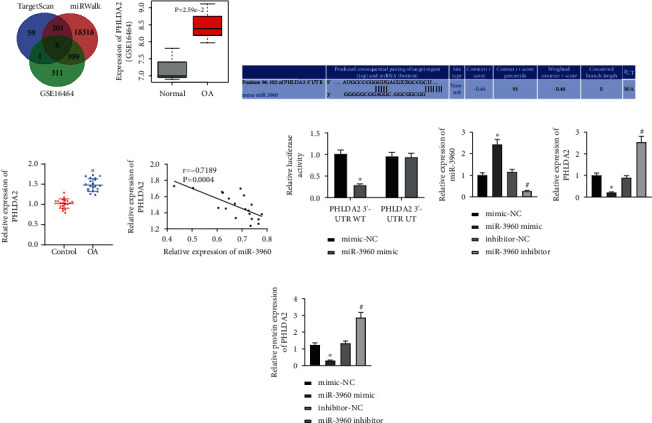
miR-3960 targets and negatively regulates PHLDA2. (a) The target gene of miR-3960 predicted using TargetScan, miRWalk, and upregulated genes in the OA-related GSE16464 dataset from GEO database. (b) PHLDA2 expression in GSE16464 dataset. (c) The binding sites between miR-3960 and PHLDA2 predicted using starBase. (d) PHLDA2 mRNA level in the cartilage tissues of OA patients (*n* = 20) and patients with traumatic amputation (*n* = 20) determined by RT-qPCR. (e) Correlation between miR-3960 and PHLDA2 analyzed by Pearson's correlation coefficient. (f) The binding of miR-3960 to PHLDA2 verified using the dual-luciferase reporter gene assay. Chondrocytes were transduced with miR-3960 mimic or miR-3960 inhibitor. (g) miR-3960 expression and PHLDA2 mRNA level in the chondrocytes determined by RT-qPCR. (h) PHLDA2 protein level in the chondrocytes determined by immunoblotting. ∗*p* < 0.05 vs. patients with traumatic amputation or chondrocytes treated with mimic-NC; ^#^*p* < 0.05 vs. chondrocytes treated with IL-1*β*; & *p* < 0.05 vs. chondrocytes treated with inhibitor-NC. Data are shown as the mean ± standard deviation of three technical replicates. Data between two groups were compared by independent *t*-test. Data comparisons among multiple groups were analyzed by the one-way ANOVA with Tukey's post hoc test.

In order to further understand the mechanism of miR-3960 affecting chondrocyte inflammation and ECM degradation in MSCs-EVs, the downstream target mRNA of miR-3960 was predicted through TargetScan and miRWalk databases, and the OA-related mRNA expression database GSE16464 was retrieved from GEO database. There were only six genes (PHLDA2, GLIS1, KCNMB3, FGF20, NRGN, and PITPNM3) found in the intersection of these three databases (Figure 3(a)). It has been reported that PHLDA2 is involved in OA [16]. Thus, PHLDA2 was selected for further experiments. In the microarray GSE16464 (Three individuals receiving autologous chondrocyte transplantation served as the control group, and PHLDA2 expression in chondrocytes in the 3 control groups and 3 patients with OA was detected) [22, 23], PHLDA2 was uncovered to be upregulated in OA samples (Figure 3(b)). There were binding sites between miR-3960 and PHLDA2 through starBase website (Figure 3(c)). RT-qPCR consistently validated an elevated PHLDA2 expression pattern in the cartilage tissues of OA patients (Figure 3(d)). The Pearson correlation analysis also suggested an inverse correlation of miR-3960 expression with PHLDA2 expression (Figure 3(e)). Dual-luciferase reporter gene assay exhibited that luciferase activity of PHLDA2 3'-UTR WT was inhibited by miR-3960 mimic with no evident difference detected in PHLDA2 3'-UTR MUT (Figure 3(f)), confirming their binding relationship. Next, chondrocytes were transduced with miR-3960 mimic or miR-3960 inhibitor, and it was experimentally determined that PHLDA2 expression was reduced in the chondrocytes transduced with miR-3960 mimic but elevated in chondrocytes transduced with the miR-3960 inhibitor (Figures 3(g) and 3(h)). The obtained data indicated that miR-3960 could target and downregulate PHLDA2.

### 3.4. miR-3960 Relieves Chondrocyte Injury by Inhibiting PHLDA2

**Figure 4:**
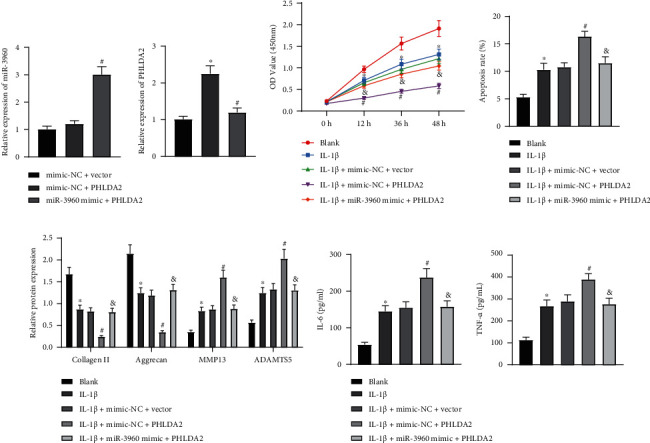
miR-3960 attenuates chondrocyte injury by inhibiting PHLDA2. Chondrocytes were transduced with miR-3960 mimic and/or PHLDA2A. (a) miR-3960 expression and PHLDA2 mRNA level in the chondrocytes determined by RT-qPCR. Chondrocytes were treated with IL-1*β* and transduced with miR-3960 mimic and/or PHLDA2A. (b) Cell proliferation assessed by CCK8 assay. (c) Apoptosis assessed by flow cytometry. (d) Protein levels of collagen II, aggrecan, MMP13, and ADAMTS5 in the chondrocytes measured by immunoblotting. (e) Levels of IL-6 and TNF-*α* in the chondrocytes measured by ELISA. ∗*p* < 0.05 vs. chondrocytes treated with mimic-NC+vector or chondrocytes without treatment; ^#^*p* < 0.05 vs. chondrocytes treated with mimic-NC+PHLDA2 or chondrocytes treated with IL-1*β*+mimic-NC+vector; & *p* < 0.05 vs. chondrocytes treated with IL-1*β*+mimic-NC+PHLDA2. Data are shown as the mean ± standard deviation of three technical replicates. Data between two groups were compared by independent *t*-test. Data comparisons among multiple groups were analyzed by the one-way ANOVA with Tukey's post hoc test, and comparison of data at different time points was analyzed using repeated measurement ANOVA with Tukey's post hoc test.

Considering the binding relationship between miR-3960 and PHLDA2, chondrocytes were transduced with mimic-NC/miR-3960 mimic or empty vector/PHLDA2 overexpression vector to assess the function of this binding affinity in chondrocyte injury. PHLDA2 was successfully overexpressed by transduction with PHLDA2 overexpression vector without affecting miR-3960 expression in chondrocytes. However, gain-of-function of miR-3960 by miR-3960 mimic contributed to elevated miR-3960 and reduced PHLDA2 expression patterns (Figure 4(a)). Furthermore, PHLDA2 elevation repressed cell proliferation and enhanced apoptosis in the IL-1*β*-treated chondrocytes, which effects were negated by further gain-of-function of miR-3960 (Figures 4(b) and 4(c)). Immunoblotting and ELISA data additionally presented that PHLDA2 elevation reduced collagen II and aggrecan levels and increased MMP13, ADAMTS5, IL-6, and TNF-*α* levels in the IL-1*β*-treated chondrocytes, while all of these changes were reversed by additional treatment of miR-3960 mimic (Figures 4(d) and 4(e)). It was, therefore, unraveled that miR-3960 could target PHLDA2 to protect chondrocytes from IL-1*β*-induced inflammation and ECM degradation.

### 3.5. PHLDA2 Upregulates SDC1 Expression

**Figure 5:**
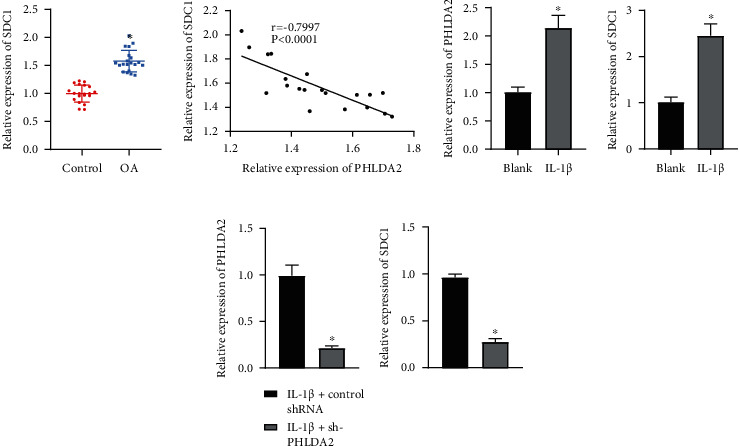
PHLDA2 elevates SDC1 expression. (a) SDC1 mRNA level in the cartilage tissues of OA patients (*n* = 20) and patients with traumatic amputation (*n* = 20) determined by RT-qPCR. (b) Correlation between PHLDA2 and SDC1 analyzed by Pearson's correlation coefficient. (c) Expression of PHLDA2 and SDC1 in IL-1*β*-induced chondrocytes determined by RT-qPCR. (d) Expression of PHLDA2 and SDC1 in IL-1*β*-induced chondrocytes transduced with sh-PHLDA2 determined by RT-qPCR. ∗*p* < 0.05 vs. patients with traumatic amputation; chondrocytes without treatment, or chondrocytes treated with IL-1*β*+control shRNA. Data are shown as the mean ± standard deviation of three technical replicates. Data between two groups were compared by independent *t*-test.

The current study has proved that miR-3960 can affect chondrocyte inflammation and ECM degradation by regulating PHLDA2 as mentioned above. It has been found that PHLDA2 can promote SDC1 expression [24]. Therefore, we speculated that PHLDA2 can affect chondrocyte inflammation and ECM degradation by promoting SDC1. RT-qPCR displayed an abundance in SDC1 expression in the cartilage tissues of OA patients (Figure 5(a)). Pearson correlation analysis revealed a positive relationship between PHLDA2 and SDC1 expression (Figure 5(b)). Both of them were further documented to be elevated in the IL-1*β*-induced chondrocytes (Figure 5(c)). Next, IL-1*β*-induced chondrocytes were transduced with sh-PHLDA2. Consequently, the loss of PHLDA2 caused a reduction of SDC1 expression in the IL-1*β*-induced chondrocytes (Figure 5(d)). It can be suggested that PHLDA2 could promote SDC1 expression.

### 3.6. SDC1 Activates Wnt/*β*-Catenin Pathway to Induce Chondrocyte Injury

**Figure 6:**
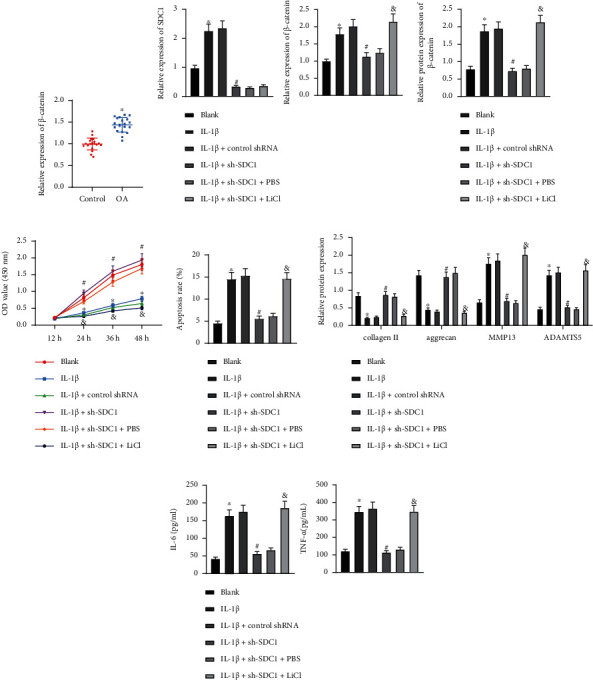
SDC1 induces chondrocyte injury by activating Wnt/*β*-catenin pathway. (a) *β*-catenin mRNA level in the cartilage tissues of OA patients (*n* = 20) and patients with traumatic amputation (*n* = 20) determined by RT-qPCR. Chondrocytes were treated with IL-1*β*, sh-SDC1, and/or LiCl. (b) mRNA levels of SDC1 and *β*-catenin in the chondrocytes determined by RT-qPCR. (c) Protein levels of SDC1 and *β*-catenin in the chondrocytes determined by immunoblotting. (d) Cell proliferation assessed by CCK8 assay. (e) Apoptosis assessed by flow cytometry. (f) Protein levels of collagen II, aggrecan, MMP13, and ADAMTS5 in the chondrocytes measured by immunoblotting. (g) Levels of IL-6 and TNF-*α* in the chondrocytes measured by ELISA. ∗*p* < 0.05 vs. patients with traumatic amputation or chondrocytes without treatment; ^#^*p* < 0.05 vs. chondrocytes treated with IL-1*β*+control shRNA; & *p* < 0.05 vs. chondrocytes treated with IL-1*β*+sh-SDC1+PBS. Data are shown as the mean ± standard deviation of three technical replicates. Data between two groups were compared by independent *t*-test. Data comparisons among multiple groups were analyzed by the one-way ANOVA with Tukey's post hoc test, and comparison of data at different time points was analyzed using repeated measurement ANOVA with Tukey's post hoc test.

Growing evidences have confirmed that SDC1 can activate Wnt/*β*-catenin pathway to promote cartilage loss, thereby facilitating OA [25–27]. Therefore, we speculate that SDC1 may promote OA by activating Wnt/*β*-catenin pathway. RT-qPCR showed that *β*-catenin expression increased in the cartilage tissues of OA patients (Figure 6(a)). Subsequently, SDC1 expression was intervened in chondrocytes which were treated with Wnt/*β*-catenin pathway agonist 20 mM LiCl in combination. RT-qPCR and Western blot analysis exhibited that shRNA-induced silencing of SDC1 downregulated the *β*-catenin expression in the IL-1*β*-treated chondrocytes, while *β*-catenin expression was rescued by additional treatment of LiCl (Figures 6(b) and 6(c)). Additionally, cell proliferation was enhanced and apoptosis was inhibited in the IL-1*β*-treated chondrocytes after SDC1 knockdown, whereas LiCl treatment reversed these changes triggered by SDC1 knockdown (Figures 6(d) and 6(e)). Immunoblotting and ELISA presented that SDC1 deficiency resulted in elevations in collagen II and aggrecan levels but reductions in MMP13, ADAMTS5, IL-6, and TNF-*α* levels in the IL-1*β*-treated chondrocytes, which effects could be compromised by LiCl (Figures 6(f) and 6(g)). The aforementioned data unveiled that SDC1 contributed to the Wnt/*β*-catenin pathway activation to aggravate chondrocyte injury.

### 3.7. miR-3960 Attenuates Chondrocyte Injury via PHLDA2/SDC1/Wnt/*β*-Catenin Axis

**Figure 7:**
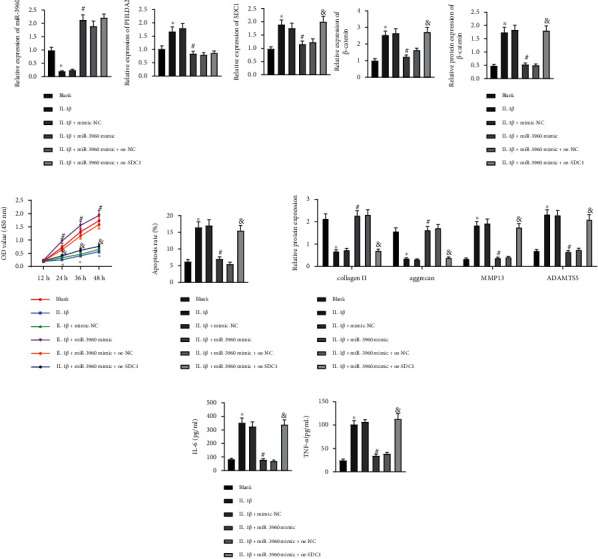
miR-3960 PHLDA2/SDC1/Wnt/*β*-Catenin axis to suppress chondrocyte inflammation and ECM degradation. Chondrocytes were treated with IL-1*β*, miR-3960 mimic, and/or oe-SDC1. (a) miR-3960 expression and mRNA levels of PHLDA2, SDC1, and *β*-catenin in chondrocytes determined by RT-qPCR. (b) Protein levels of PHLDA2, SDC1, and *β*-catenin in chondrocytes determined by immunoblotting. (c) Cell proliferation assessed by CCK8 assay. (d) Apoptosis assessed by flow cytometry. (e) Protein levels of collagen II, aggrecan, MMP13, and ADAMTS5 in chondrocytes measured by immunoblotting. (f) Levels of IL-6 and TNF-*α* in chondrocytes measured by ELISA. ∗*p* < 0.05 vs. chondrocytes without treatment; ^#^*p* < 0.05 vs. chondrocytes treated with IL-1*β*+mimic-NC; & *p* < 0.05 vs. chondrocytes treated with IL-1*β*+miR-3960 mimic+oe-NC. Data are shown as the mean ± standard deviation of three technical replicates. Data between two groups were compared by independent *t-*test. Data comparisons among multiple groups were analyzed by the one-way ANOVA with Tukey's post hoc test, and comparison of data at different time points was analyzed using repeated measurement ANOVA with Tukey's post hoc test.

We intervened miR-3960 and/or SDC1 expression in the IL-1*β*-exposed chondrocytes and found that the increase of miR-3960 expression resulted in diminished expression of PHLDA2, SDC1, and *β*-catenin in the IL-1*β*-treated chondrocytes, while further restoration of SDC1 did not change the expression of miR-3960 and PHLDA2 while upregulating *β*-catenin levels (Figures 7(a) and 7(b)). In response to miR-3960 gain-of-function, cell proliferation was boosted and apoptosis repressed in the IL-1*β*-treated chondrocyte, while overexpression of SDC1 counteracted the pro-proliferative and antiapoptotic effects of miR-3960 (Figures 7(c) and 7(d)). Meanwhile, collagen II and aggrecan levels increased and MMP13, ADAMTS5, IL-6, and TNF-*α* levels decreased by miR-3960 mimic in the IL-1*β*-treated chondrocytes, while the induced changes were reversed by the additional manipulation with oe-SDC1 (Figures 7(e) and 7(f)). Hence, it was to that miR-3960 suppressed PHLDA2 expression to downregulate SDC1 whereby ameliorating chondrocyte injury.

### 3.8. MSCs-EVs Carrying miR-3960 Relieves Chondrocyte Injury

**Figure 8:**
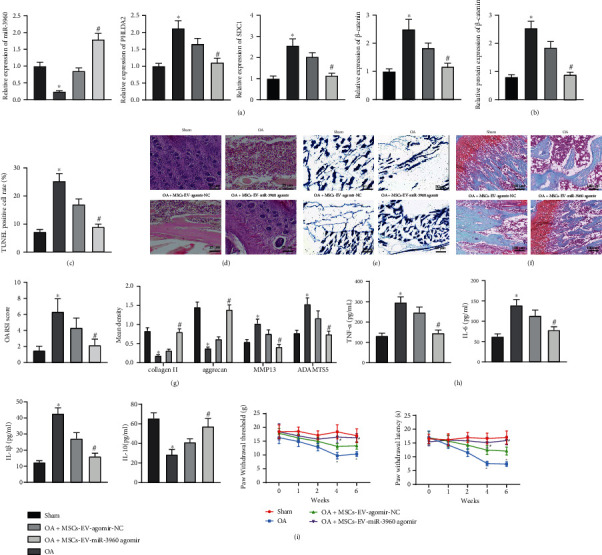
MSCs-EVs-encapsulated miR-3960 relieves chondrocyte inflammation and ECM degradation in OA mice. OA mouse models were established and injected with MSCs-EVs-miR-3960 agomir (*n* = 6). (a) miR-3960 expression and mRNA levels of PHLDA2, SDC1, and *β*-catenin in chondrocytes of OA mice determined by RT-qPCR. (b) Protein levels of PHLDA2, SDC1, and *β*-catenin in chondrocytes of OA mice determined by immunoblotting. (c) Apoptosis of chondrocytes in OA mice detected by TUNEL assay (200 ×). (d) Articular cartilage injury of OA mice assessed by HE staining (scale bar = 25 *μ*m). (e) aggrecan content of cartilage tissues in OA mice detected by alcian blue staining (scale bar = 50 *μ*m). (f) ECM degradation of cartilage tissues in OA mice assessed by Safranin O-Fast Green staining (scale bar = 100 *μ*m) and OARSI score. (g) Protein levels of collagen II, aggrecan, MMP13, and ADAMTS5 in chondrocytes of OA mice measured by immunohistochemistry. (h) Levels of IL-6 and TNF-*α* in the serum of OA mice measured by ELISA. (i) PWT and PWL of mice. ∗*p* < 0.05 vs. sham-operated mice, #*p* < 0.05 vs. OA mice injected with MSCs-EVs-agomir-NC. Data are shown as the mean ± standard deviation of three technical replicates. Data between two groups were compared by independent *t*-test. Data comparisons among multiple groups were analyzed by the one-way ANOVA with Tukey's post hoc test.

OA mouse models were established for validation, and OA mice were injected with MSCs-EVs-mimic-NC/MSCs-EVs-miR-3960 mimic. RT-qPCR and immunoblotting analyses presented that relative to the sham-operated mice, miR-3960 expression decreased while that of PHLDA2, SDC1, and *β*-catenin increased in the mice induced with OA-like pathology. MSCs-EVs-agomir-NC treatment rescued miR-3960 expression, resulting in diminished PHLDA2, SDC1, and *β*-catenin levels, and MSCs-EVs-miR-3960 agomir induced more significant effects (Figures 8(a) and 8(b)). TUNEL assay exhibited that MSCs-EVs-agomir-NC could delay the OA-induced apoptosis of chondrocytes, and that MSCs-EVs-miR-3960 agomir exerted a superior antiapoptotic effect (Figure 8(c)).

HE staining showed smooth cartilage surface, healthy synovium, no loose body, and neat edge of articular cartilage along with regularly arranged chondrocytes in normal mice. After the successful OA induction, rough and dry cartilage surface, thin and rough superficial cartilage with many cracks on the cartilage surface, many loose bodies of different sizes in the joint, unclear boundary of articular cartilage, and disorderly arranged chondrocytes accompanied by loss of chondrocytes were visualized. Strikingly, MSCs-EVs-agomir-NC was witnessed to attenuate the progressive OA-like pathology, contributing to slightly rough bone surface, relieved cracks on cartilage surface, and improvement in disordered arrangement of chondrocytes. Furthermore, MSCs-EVs-miR-3960 agomir treatment induced changes in the mice rendered with OA, such as less smooth cartilage surface, no loose body, and less neat edge of articular cartilage, suggesting a superior ameliorative effect of MSCs-EVs-miR-3960 on OA (Figure 8(d)).

Moreover, reduced aggrecan content and serious degradation of ECM were noticed in OA mice. MSCs-EVs-miR-3960 agomir rescued the aggrecan content and relieved degradation of ECM in the OA modeled mice, exhibiting a superior effect than MSCs-EVs-agomir-NC (Figures 8(e) and 8(f)). As shown by the OARSI grading system, the OA-modeled mice presented an elevated OARSI score of the knee joint tissues, while MSCs-EVs-miR-3960 agomir markedly reduced the OARSI score as compared to MSCs-EVs-agomir-NC (Figure 8(f)).

Immunohistochemistry and ELISA revealed that relative to sham-operated mice, collagen II, aggrecan, and IL-10 levels were decreased and MMP13, ADAMTS5, IL-1*β*, IL-6, and TNF-*α* levels elevated in the OA-modeled mice. However, the changes in the abovementioned anabolic, catabolic, proinflammatory, and anti-inflammatory markers in the OA context were reversed in by MSCs-EVs-agomir-NC, which effects were more significant after the treatment with MSCs-EVs-miR-3960 agomir (Figures 8(g) and 8(h)). To evaluate the effect of MSCs-EVs on pain recovery, we experimentally measured the PWT and PWL values and found that PWT and PWL exhibited no significant change in the sham-operated mice compared with the baseline PWT and PWL values (prior to the modeling). In comparison to the sham-operated mice, reduced PWT and PWL values were observed in the OA-modeled mice, while MSCs-EVs-miR-3960 agomir exerted a more significant promotive effect on the PWT and PWL values in the OA-modeled mice (Figure 8(i)). It can be concluded that miR-3960 shuttled by MSCs-EVs attenuated the severity of OA.

## 4. Discussion

OA is becoming more prevalent with the combined effects of aging, increasing obesity, and the increasing number of joint injuries [2]. It is the main reason for disability in the aged, resulting in pain, loss of function, and reduced quality of life [28]. MSC-based therapy can promote cartilage regeneration and repair, contributing to its therapeutic efficacy in rabbits [29] and OA patients [30]. In recent years, the efficacy of MSC-derived EVs in OA has attracted great attention [5]. Also, secretomes originated from MSCs have emerged as anti-inflammatory mediators in rat chondrocytes [31]. More importantly, MSC-derived EVs exert potential to accelerate cartilage regeneration and osteogenesis through paracrine mechanisms due to their ability to transfer molecules such as mRNAs and miRNAs [32, 33]. Albanese et al. demonstrated that EV-derived miRNAs do not act as effectors of cell-to-cell communication [34], but other studies have proved that MSCs-EVs contain various miRNAs and can deliver miRNAs to other target cells [35–37]. Although it has been reported that MSC-EVs most probably work through the protein rather than the RNA [38], more previous studies suggested that EVs can cause the biological effects of recipient cells by carrying mRNAs and miRNAs and delivering them to target cells, then eliciting a biological response in recipient cells [39–41]. However, most RNAs in EVs are short with a length between 200 and 400 nts, and the average length of mRNAs in humans is greater than 2000 nts, so RNAs in EVs are often too short to carry protein-coding information [42]. Therefore, the therapeutic effects of MSC-EVs are increasingly attributed to the cell-to-cell transfer of miRNAs [43]. Here, we found that miR-3960 shuttled by MSC-derived EVs could be transferred into chondrocytes, where miR-3960 exerted inhibited properties in chondrocyte injury in OA through the downregulation of PHLDA2/SDC1/Wnt/*β*-catenin axis.

Initially, the obtained data revealed that MSC-derived EVs inhibit chondrocyte injury and ameliorate inflammation in the OA context. The roles of EVs in OA have been demonstrated [7]. Consistently, a recent study has confirmed the promoting role of BMSC-derived EVs in cartilage repair and extracellular matrix synthesis to relieve knee pain in OA [44]. It is known that apoptosis and inflammation of chondrocytes serve as hallmarks for progression of OA, and alleviation of chondrocyte apoptosis and inflammation contributes to inhibition of OA [3]. MSC-derived EVs carrying miRNAs have been unveiled to promote proliferation and inhibit apoptosis of chondrocytes to attenuate chondrocyte injury in OA [45], which is consistent with our findings. For instance, the alleviative effect of MSC-derived EVs on OA is expounded achieved by delivery of miRNAs such as miR-140-5p [46]. In the present study, we also revealed that miR-3960 shuttled by MSCs-EVs boosted cell proliferation and resistance to apoptosis in chondrocytes, whereby alleviating chondrocyte injury in OA. Furthermore, miR-3960 shuttled by MSCs-EVs carrying miR-3960 could not only restrain ECM degradation, as evidenced by elevated levels of collagen II and aggrecan and reduced levels of MMP13, ADAMTS5, but also limit the release of proinflammatory proteins (IL-6 and TNF-*α*) in chondrocytes. Multiple miRNAs are dysregulated in inflammatory disorders in OA [4]. A recent study has elaborated the relation between inflammation and OA pain and prognosis, and that new appealing therapies targeting inflammation might contribute to satisfactory pain relief [47]. A large body of evidence has verified that activation of inflammatory factors, including IL1*β*, TNF-*α*, and IL-6 [48], is implicated in the development and progression of and OA [49, 50], while the blockage of these proinflammatory cytokines serves as an appealing treatment option for OA [51]. Moreover, elevation of IL-1*β* can be observed in synovial fluid, subchondral bone, and cartilage tissues from OA patients [52]. It is widely accepted that IL-1*β* is involved in the destruction of articular cartilage due to induction of MMPs and ADAMTS, and suppression of IL-1*β*-induced ECM-degrading enzyme syntheses and secretion results in the chondrocyte ECM breakdown and OA development [53]. Accumulating evidences have demonstrated that multiple miRNAs, such as miR-335-5p, miR-152, and miR-93, are downregulated in OA chondrocytes, and reexpression of miRNAs delay OA progression through enhancing viability, suppressing apoptosis of chondrocytes, and attenuating the inflammatory response with a suppressive effect on TNF-*α*, IL-1*β*, and IL-6 [3, 13, 54]. Another study has also proved that elevated miR-140 elevates collagen II expression and reduces MMP-13 and ADAMTS-5 expressions to inhibit ECM degradation, thereby alleviating OA progression [55]. The effects of miR-3960 on osteoblastogenesis have been explored [56], while its protective role in chondrocyte injury in OA is newly identified here.

In addition, we experimentally testified that miR-3960 targeted and inhibited PHLDA2 to attenuate chondrocyte injury. PHLDA2 has been demonstrated to participate in the progression of OA [16]. Moreover, elevated expression of PHLDA2 is related to poor prognoses in several types of cancer, and PHLDA2 could upregulate SDC1 expression, which contributes to cancer cell aggressiveness [24]. It is striking in the present study to note that PHLDA2 can relieve chondrocyte injury in OA by increasing SDC1. A recent study has uncovered that overexpressed SDC1 leads to aggravation of inflammation and OA-like damage, and that MSC-derived exosomal miR-9-5p exerts anti-inflammatory and chondroprotective effects on OA by inhibiting SDC1 expression [57]. Furthermore, the data in this study illustrated that SDC1 could activate the Wnt/*β*-catenin pathway to induce chondrocyte injury. Wnt/*β*-Catenin pathway is activated in the setting of OA, which promotes the differentiation of proliferating chondrocytes into hypertrophic chondrocytes to prevent cartilage degradation in a mouse model of OA [58]. Furthermore, the Wnt/*β*-catenin pathway activates inflammatory factors by regulating cartilage ECM degradation mediated by MMPs and aggrecanases [26]. IL-1*β* could activate Wnt/*β*-catenin signaling to induce chondrocyte apoptosis, suggesting that the inhibited Wnt/*β*-catenin pathway may repress IL-1*β*-induced cartilage degradation [59]. The aforementioned findings collectively supported the notion that miR-3960 shuttled by MSC-secreted EVs attenuated OA-related chondrocyte injury by inhibiting the PHLDA2/SDC1/Wnt/*β*-catenin axis, which was addressed in this study.

## 5. Conclusions

**Figure 9:**
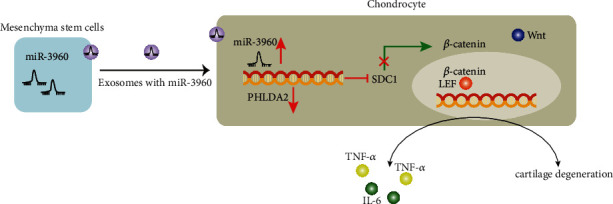
MSCs-EVs carrying miR-3960 inhibits PHLDA2 to suppress SDC1 and the Wnt/*β*-catenin pathway activation, thereby repressing the chondrocyte injury in OA.

Our study accumulatively demonstrated that the transfer of miR-3960 via MSC-derived EVs altered the SDC1/Wnt/*β*-catenin expression, by negatively regulating PHLDA2, which in turn alleviates chondrocyte injury in OA (Figure 9). Our findings pave way for the development of effective therapeutic strategies for inhibiting chondrocyte injury in OA. However, the clinical significance of miR-3960 shuttled by MSC-derived EVs and PHLDA2 as well as their interaction in the chondrocyte injury in the progression of OA should be further investigated.

## Data Availability

The datasets used and/or analyzed in the present study are available from the corresponding author on reasonable request.

## Abbreviations

OA: Osteoarthritis
MSCs: Mesenchymal stem cells
EVs: Extracellular vesicles
3'UTR: 3′ untranslated region
DMEM: Dulbecco's Modified Eagle's Medium
FBS: Fetal bovine serum
HE: Hematoxylin & eosin
PBS: Phosphate buffer saline
NC: Negative control
BMSCs: Bone marrow-derived MSCs
RIPA: Radio immunoprecipitation assay
BCA: Bicinchoninic acid
NTA: Nanoparticle tracking analysis
OD: Optical density
PCR: Polymerase chain reaction
IgG: Immunoglobulin G.

## Supplementary Materials

Supplementary 1 Figure S1: identification of chondrocytes. A, Morphology of primary cultured mouse chondrocytes after adhesion under the microscope (scale bar =100 *μ*m). The isolated primary chondrocytes were spherical and suspended before adherent. The primary chondrocytes partially adhered to the wall after incubation for 9-12 h, and most of them adhered at 24 ho. Some cells began to extend, and the cell volume was small and polygonal. B, The expression of type II collagen in primary cultured mouse chondrocytes (scale bar = 25 *μ*m). The type II collagen is rich in cartilage matrix. The synthesis and secretion of type II collagen can be used as a specific indicator for chondrocytes to maintain their differentiation phenotype. The type II collagen secreted by cultured chondrocytes was red and the nucleus was blue under fluorescence microscope. The typical morphology of chondrocytes could be seen after the synthesis of cytoplasm and nucleus. C, Representative images of Toluidine blue staining. The cytoplasm of primary cultured mouse chondrocytes was light blue, the nucleus was dark blue and clear. The extracellular matrix was light blue (scale bar = 25 *μ*m).

Supplementary 2 Table S1: RT-qPCR primer sequences. Note: RT-qPCR, reverse transcription quantitative polymerase chain reaction; F, forward; R, reverse.
