# Supplementary material for: miR-3960 from Mesenchymal Stem Cell-Derived Extracellular Vesicles Inactivates SDC1/Wnt/β-Catenin Axis to Relieve Chondrocyte Injury in Osteoarthritis by Targeting PHLDA2
Source: Stem Cells Int. 2022 Aug 25;2022:9455152. doi: 10.1155/2022/9455152 (PMC9438433; doi:10.1155/2022/9455152)
Supplement: Supplementary 1 — Figure S1: identification of chondrocytes. A, Morphology of primary cultured mouse chondrocytes after adhesion under the microscope (scale bar =100 μm). The isolated primary chondrocytes were spherical and suspended before adherent. The primary chondrocytes partially adhered to the wall after incubation for 9-12 h, and most of them adhered at 24 ho. Some cells began to extend, and the cell volume was small and polygonal. B, The expression of type II collagen in primary cultured mouse chondrocytes (scale bar = 25 μm). The type II collagen is rich in cartilage matrix. The synthesis and secretion of type II collagen can be used as a specific indicator for chondrocytes to maintain their differentiation phenotype. The type II collagen secreted by cultured chondrocytes was red and the nucleus was blue under fluorescence microscope. The typical morphology of chondrocytes could be seen after the synthesis of cytoplasm and nucleus. C, Representative images of Toluidine blue staining. The cytoplasm of primary cultured mouse chondrocytes was light blue, the nucleus was dark blue and clear. The extracellular matrix was light blue (scale bar = 25 μm). [file 9455152.f1.doc]

**
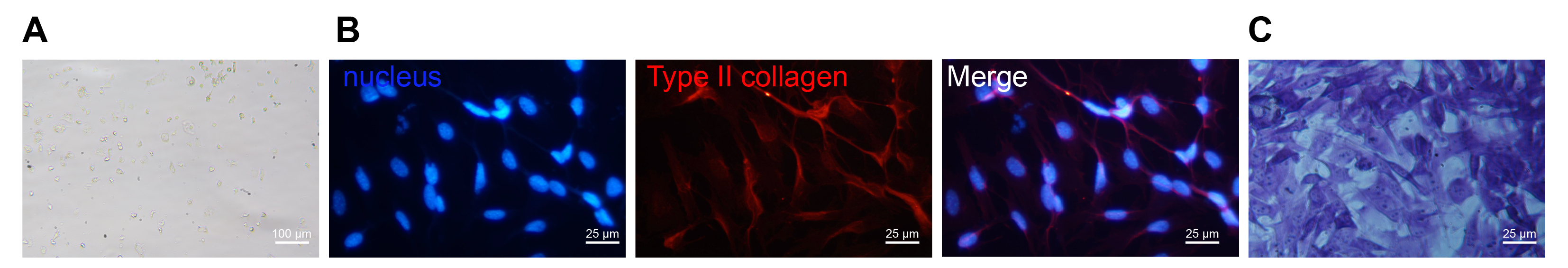
**

**FIGURE S1** Identification of chondrocytes.

A, Morphology of primary cultured mouse chondrocytes after adhesion under the microscope (scale bar = 100 μm). The isolated primary chondrocytes were spherical and suspended before adherent. The primary chondrocytes partially adhered to the wall after incubation for 9-12 h, and most of them adhered at 24 ho. Some cells began to extend, and the cell volume was small and polygonal. B, The expression of type II collagen in primary cultured mouse chondrocytes (scale bar = 25 μm). The type II collagen is rich in cartilage matrix. The synthesis and secretion of type II collagen can be used as a specific indicator for chondrocytes to maintain their differentiation phenotype. The type II collagen secreted by cultured chondrocytes was red and the nucleus was blue under fluorescence microscope. The typical morphology of chondrocytes could be seen after the synthesis of cytoplasm and nucleus. C, Representative images of Toluidine blue staining. The cytoplasm of primary cultured mouse chondrocytes was light blue, the nucleus was dark blue and clear. The extracellular matrix was light blue (scale bar = 25 μm).
