# Supplementary material for: miR-3960 from Mesenchymal Stem Cell-Derived Extracellular Vesicles Inactivates SDC1/Wnt/β-Catenin Axis to Relieve Chondrocyte Injury in Osteoarthritis by Targeting PHLDA2
Source: Stem Cells Int. 2022 Aug 25;2022:9455152. doi: 10.1155/2022/9455152 (PMC9438433; doi:10.1155/2022/9455152)
Supplement: Supplementary 2 — Table S1: RT-qPCR primer sequences. Note: RT-qPCR, reverse transcription quantitative polymerase chain reaction; F, forward; R, reverse. [file 9455152.f2.doc]

**TABLE S1** RT-qPCR primer sequences

| Genes | Primer sequence |
| --- | --- |
| miR-3960 (*mouse*) | F: 5ʹ-GGCGGCGGCGGAGGCGGGGG-3’ |
|  | R: Universal reverse primer |
| miR-3960 (*Homo*) | F: 5ʹ-GGCGGCGGCGGAGGCGGGGG-3’ |
|  | R: Universal reverse primer |
| U6 | F: 5ʹ-CTCGCTTCGGCAGCACA-3’ |
|  | R: 5ʹ-AACGCTTCACGGAATTTGCGT-3’ |
| syn-cel-miR-39 | F: 5′-GGGGAGCTGATTTCGTCTTG-3′ |
|  | R: 5′-CTCAACTGGTGTCGTGGAGT-3′ |
| SDC1 (*mouse*) | F: 5′-GAGGAGACAGAGCCTAACGC-3′ |
|  | R: 5′-AGAGTCATCCCCAGAGCCAT-3′ |
| SDC1 (*Homo*) | F: 5′-CGAGCTGAAAGGCCGGGAA-3′ |
|  | R: 5ʹ-CAGGGGTTGAGGTCTCATGG-3’ |
| β-catenin (*mouse*) | F: 5′-GGCGGCCGCGAGGTA-3′ |
|  | R: 5′-TTAGTGGGATGAGCAGCGTC-3′ |
| PHLDA2 (*mouse*) | F: 5′-AAACCGTGAAGACCTCCGAC-3′ |
|  | R: 5′-CAATAAGTTAGCGCACCCGC-3′ |
| PHLDA2 (*Homo*) | F: 5′-ACAAGGAGATCGACTTCCGC-3′ |
|  | R: 5′-CCACAGCCGGATGGTAGAAA-3′ |
| GAPDH (*mouse*) | F: 5′-CCCTTAAGAGGGATGCTGCC-3’ |
|  | R: 5′-ACTGTGCCGTTGAATTTGCC-3’ |
| GAPDH (*Homo*) | F: 5′-GGTCACCAGGGCTGCTTTTA-3’ |
|  | R: 5′-CCCGTTCTCAGCCATGTAGT-3’ |

Note: RT-qPCR, reverse transcription quantitative polymerase chain reaction; F, forward; R, reverse.
